# P01.39. Synergism of herbs in classical Chinese medicine: evidence from HPLC

**DOI:** 10.1186/1472-6882-12-S1-P39

**Published:** 2012-06-12

**Authors:** K Shaw, K Wright, J Wang, P Kalnins

**Affiliations:** 1 National College of Natural Medicine, Portland, USA

## Purpose

Classical Chinese herbal therapy uses herb combinations within formulas based upon the belief that there is a synergistic effect between herbs. Herbal pairs and combinations may change the hydrophobicity and ion concentration of the decoction. These changes may increase extraction of constituents from other herbs within the formula. Furthermore, herbs may react within the decoction medium to create new chemical structures. The purpose of this study is to compare chemical signatures of individual Chinese herbs with paired herbal formulas, as they are used in Classical Chinese medicine.

## Methods

This study investigates the synergism of licorice with two herbs (Bupleurum chinense and Zingiberis officinalis) commonly used in Classical Chinese Medicine. Each sample was decocted in de-ionized water for one hour at 100 °C. Individual and paired decoctions were analyzed using High Performance Liquid Chromatography (HPLC) to evaluate differences in chemical signatures between extractions.

## Results

Chemical constituent concentration varied between single herb and paired herb decoctions. In the paired decoctions, many chemical peaks increased in concentration. Interestingly, other peaks decreased in concentration, though none disappeared.

## Conclusion

This preliminary data demonstrate that paired herbal decoctions contain increased levels of active components. Furthermore, a decrease in peaks within the paired decoction but not in the individual formulas suggests that herb decoctions generated new chemical structures. This study represents foundational research into synergistic relationships between herbs.

